# Highly Stable Potentiometric (Bio)Sensor for Urea and Urease Activity Determination

**DOI:** 10.3390/membranes11110898

**Published:** 2021-11-20

**Authors:** Marcin Urbanowicz, Kamila Sadowska, Agnieszka Paziewska-Nowak, Anna Sołdatowska, Dorota G. Pijanowska

**Affiliations:** Nalecz Institute of Biocybernetics and Biomedical Engineering, Polish Academy of Sciences, Ks. Trojdena 4, 02-109 Warsaw, Poland; ksadowska@ibib.waw.pl (K.S.); apaziewska@ibib.waw.pl (A.P.-N.); asoldatowska@ibib.waw.pl (A.S.); dpijanowska@ibib.waw.pl (D.G.P.)

**Keywords:** potentiometric biosensor, urease activity determination, urea determination, polyazulene, electroconducting polymers, intermediate transducing layer, ammonium ion selective electrode, solid contact, ion selective membranes

## Abstract

There is growing interest for bioanalytical tools that might be designed for a specific user, primarily for research purposes. In this perspective, a new, highly stable potentiometric sensor based on glassy carbon/polyazulene/NH_4_^+^-selective membrane was developed and utilized for urease activity determination. Urease–urea interaction studies were carried out and the Michaelis–Menten constant was established for this enzymatic reaction. Biofunctionalization of the ammonium ion-selective sensor with urease lead to urea biosensor with remarkably good potential stability (drift coefficient ~0.9 mV/h) and short response time (t_95%_ = 36 s). The prepared biosensor showed the Nernstian response (S = 52.4 ± 0.7 mV/dec) in the urea concentration range from 0.01 to 20 mM, stable for the experimental time of 60 days. In addition, some insights into electrical properties of the ion-to-electron transducing layer resulting from impedance spectroscopy measurements are presented. Based on the RCQ equivalent circuits comparison, it can be drawn that the polyazulene (PAz) layer shows the least capacitive behavior, which might result in good time stability of the sensor in respect to response as well as potential E^0^. Both the polyazulene-based solid-contact ion selective electrodes and urea biosensors were successfully used in trial studies for determination of ammonium ion and urea in human saliva samples. The accuracy of ammonium ion and urea levels determination by potentiometric method was confirmed by two reference spectrophotometric methods.

## 1. Introduction

The study of enzyme activity is one of the most important biochemical analysis, in both medical diagnosis and in treatment. Understanding how enzymes work and how their activity can be regulated is a key study providing insight into disease mechanisms and development of many pharmaceuticals. Enzyme activity assays are also routinely performed in research on cell and tissue engineering, as well as in environmental studied. This induces a growing interest for bioanalytical tools that might be designed for a specific user, primarily for research purposes. In the research conducted with our co-workers on various cell systems, including cells with restored urea cycle, we have observed a need for a stable and reproducible analytical tool for urea determination. Most of literature on the urease activity determination proposes spectrophotometric readout of various indicators [1,2,3,4,5]. To the best of our knowledge, electrochemical sensors are rarely used for measurements of urease activity. The voltammetric sensor [6] and conventional potentiometric cationic glass electrode [7] have been reported. Contrarily, potentiometric sensors are commonly used for urea determination in biological and environmental samples [8,9,10]. Direct potentiometric sensing with minimal sample pre-treatment and fast and reliable data processing is the method-of-choice in medical laboratories for determination of many diagnostically important analytes, for example, Na^+^, K^+^, Ca^2+^, H^+^, Cl^−^, and SCN^−^ [10,11,12,13,14]. Enzyme immobilization on the surface of the ion-selective membrane enables detecting and determining the non-ionic analytes, such as urea, by detecting changes in the concentration of ionic products of enzymatic reactions. In the case of urea biosensors, urease is used as a bioreceptor, and the hydrolysis products, namely H^+^ [15,16] and NH_4_^+^ [17,18], are determined, whose ionic form is assured using an appropriate buffer. 

Although the potentiometric sensors have been studied for decades, their breakthrough resulted from the progress in the field of materials science has only recently been observed. The use of new materials, as conductive polymers or nanostructures can improve the detection limit, biocompatibility, and potentiometric (bio)sensor stability [19,20,21,22,23,24,25,26,27,28,29,30,31]. To improve the metrological parameters of potentiometric urea sensors, conductive polymers, such as polypyrrole (PPy) [22,23], poly(*o*-phenylenediamine) [24], polyaniline [25,26,27], and poly(3-hexylthiophene-*co*-3-thiopheneacetic acid) copolymer [28], have applied. Although the profits of their usage in potentiometric sensors are indisputable, such constructions are not flawless. The sensors based on PPy deposited on ITO [22], despite a wide measuring range, had a relatively long response time (70–90 s), while the sensor based on polypyrrole/polyethyleneimine hybrid film [23], despite a satisfactory sensitivity and response time, had a narrow measuring range (0.5–10 mM). More recent studies have shown that usage of hydrophobic conducting polymers as solid-contacts (SC) for ion-selective membrane (ISM) has a beneficial effect on the sensor performance. Guziński et al. reported, that highly hydrophobic PEDOT-C_14_ prevents the detachment of the lipophilic ISM from the SC and the accumulation of an aqueous layer at their interface [29]. Electropolymerized hydrophobic polyazulene (PAz) has revealed similar behavior, minimizing the water uptake between the ISM and the SC layer [30,31]. 

Considering the recent advances in the potentiometry and the specific analytical needs of research conducted in our laboratory, a highly stable solid-contact potentiometric (bio)sensor for urease activity and urea determination has been proposed. Urease activity and the Michaelis–Menten constant for enzymatic reaction of urea deamination was estimated. In addition, developed polyazulene-based solid-contact ammonium ion-selective electrodes and their urease functionalized analogues were trialled for NH_4_^+^ and urea determination in this study.

## 2. Materials and Methods

### 2.1. Chemicals

Nonactin (ammonium ionophore I—Sigma–Aldrich, Darmstadt, Germany) was used to obtain NH_4_^+^ selective membranes. Poly(vinyl chloride) (PVC of high molecular weight), poly(vinyl chloride) carboxylated 1.8%, 5% and 10%, bis(2-ethylhexyl) sebacate (DOS) ≥ 97%, potassium tetrakis(*p*-chlorophenyl)borate (KTpClPhB) ≥ 97%; and potassium tetrakis [3,5-bis(trifluoromethyl)phenyl]borate (KTpFPhB) were all purchased from Sigma–Aldrich (Darmstadt, Germany). For preparation of calibration solutions NaCl ≥ 99.9%, KCl ≥ 99.5%, CaCl_2_·6H_2_O ≥ 99%, MgCl_2_·6H_2_O ≥ 99.5%, NH_4_Cl ≥ 99.5%, MnCl_2_·4H_2_O ≥ 99%, KOH, methanol, and pH buffer (5.00 ± 0.05, 7.00 ± 0.05, 9.00 ± 0.05), from Avantor Performance Materials, Poland S.A. (Gliwice, Poland) were used. For electrodes surface modification: 3,4-ethylenedioxythiophene (EDOT) ≥ 97%, poly(sodium 4-styrenesulfonate) (PSS) average M_w_ ~1,000,000, azulene (Az) ≥ 99%, and tetrabutylammonium hexafluorophosphate (TBAPF_6_) ≥ 99% were all purchased from Sigma–Aldrich (Darmstadt, Germany). For biosensor preparation and calibration: urease (EC 3.5.1.5) from *Canavalia ensiformis* 63,163 U/g solid, urea ≥ 98%, albumin bovine serum (BSA) ≥ 98%, glutaraldehyde solution 25% in H_2_O (GA), acetonitrile (ACN) ≥ 99.8%, *N*-(3-dimethylaminopropyl)-*N′*-ethylcarbodiimide hydrochloride (EDC) ≥ 98%, and *N*-hydroxysuccinimide (NHS) ≥ 98% were purchased from Sigma–Aldrich (Darmstadt, Germany) and tris(hydroxymethyl)aminomethane ≥ 99% was purchased from POL-AURA (Warsaw, Poland). For colorimetric tests QuantiChrom™ Urea Assay Kit (Hayward, NJ, USA) was used. Sodium nitroprusside, sodium hypochlorite solution (10–15% chlorine) were purchased in Sigma–Aldrich (Darmstadt, Germany); disodium versenate dehydrate (EDTA), sodium salicylate, sodium dihydrogen phosphate, and disodium hydrogen phosphate were purchased from Avantor Performance Materials, Poland S.A. (Gliwice, Poland). All reagents were of analytical grade. A freshly deionised water obtained by the reverse osmosis (RO) MiliQ station (18.2 MΩ) was used.

### 2.2. Instrumentation

All measurements were carried out at 25 °C. Data from potentiometric measurements were recorded using a 16-channel precision electrochemistry interface EMF16 Lawson Labs Inc. (Malvern, PA, USA). For the preparation of ammonium-selective sensors glassy carbon, gold and platinum electrodes with a diameter of 2 mm purchased from Mineral (Lomianki, Poland) were used. The reference electrode was Orion™ ROSS Ultra™ 800500U (Waltham, MA, USA). The CX-705 Elmetron pH meter (Zabrze, Poland) was used to measure the pH of the prepared solutions and saliva samples.

The PalmSens4 system (Houten, The Netherlands) was used for coating of electrodes with the polyazulene layer using cyclic voltammetry method. For galvanostatic coating of electrodes with the PEDOT:PSS layer, Solartron impedance analyzer SI 1260 (Elgin, IL, USA) with an electrochemical interface SI 1287 was used.

For electrochemical impedance analysis of GC, GC/PAz and GC/PEDOT:PSS electrodes, potentiostat/galvanostat with impedance analyzer PalmSens4 system was used. The fixed-spaced three electrode system consisted of a double junction Ag/AgCl reference electrode, a platinum counter electrode and a GC based working electrode. Prior measurements the electrodes were conditioned in 10 mM KCl solution for 15 min. The impedance measurements were performed in frequency range from 100 Hz to 1 MHz with AC amplitude of 50 mV and a zero DC offset.

In the case of colorimetric analysis microplate spectrophotometer Synergy HT (BioTEK, Winooski, MA, USA) was used.

A static contact angle of electrode surface was measured using a goniometer DSA25, KRŰSS GmbH (Hamburg, Germany). SEM TM-1000 Hitachi (Ramsey, NJ, USA) was used to study morphology of PAz and PEDOT layers were deposited on carbon electrodes.

### 2.3. ISEs Preparation

The surface of electrodes was polished with fine-grained abrasive paper 1200, 2000, 4000, and Al_2_O_3_ with a grain size of 0.1 μm. Then, the electrodes were consecutively immersed in 1 M KOH/methanol solution, deionized water, 1 M HNO_3_ and again in deionized water. A PEDOT:PSS layer was deposited on the electrode surface from an aqueous solution of 0.015 M of EDOT monomer and 0.1 M of NaPSS supporting electrolyte. The polymer was deposited on the working electrodes using a galvanostatic method with current density of 0.2 mA/cm^2^ and process time 714 s [32]. The electrodes were then dried using compressed air for 15 min. Azulene was electrochemically polymerized on the electrodes surface. The polymerization was performed according to the procedure provided in Lindfor’s work [30,31]. Polyazulene films were formed by cycling the potential between −0.6 V and 1.2 V (10 cycles, ν = 50 mV/s) in ACN solutions containing 0.01 M azulene and 0.1 M TBAPF_6_. Prior to the ISM deposition, the PAz solid-contacts were p-doped by polarization at 0.2 V for 5 min in a 0.1 M TBAPF_6_/ACN solution.

The NH_4_^+^−ion selective membrane was composed of 0.2 wt.% potassium tetrakis[3,5-bis(trifluoromethyl)phenyl]borate, 1 wt.% nonactin, 66.8 wt.% bis-(2-ethylhexyl)-sebacate, and 32 wt.% PVC (ISM_I_) or PVC-COOH (ISM_II_). In ISM_III_ and ISM_IV_, KTpFPhB was excluded or replaced by KTpClPhB, respectively. More details concerning the study on ISM composition of manufactured ISEs can be found in Table S1 in Supplementary materials. The dried electrodes with PEDOT:PSS or PAz layer were modified by dropping 30 μL of the ion-selective membrane cocktail. After 24 h required for drying, the electrodes were conditioned in a solution of 1 mM NH_4_Cl for next 24 h.

Manufactured ISEs were compared in terms of metrological parameters such as sensitivity, selectivity and linear range and the results are summarized in Table S2 and presented in Figure S1 in Supplementary materials.

### 2.4. Biofunctionalization of ISEs

Sensors with ISM_I_ were biofunctionalized with urease. Solutions of both urease and BSA with different concentration ratios were prepared. In the first step of biofunctionalization, 20 μL of urease and BSA solution was deposited on the surface of ion-selective membranes. After 2 h, deposition of the proteins was repeated. Then, 20 µL of 5% GA solution was dropped and left at 4 °C for 2 h in order to cross-link the previously deposited proteins. Finally, the electrodes were rinsed with deionised water and conditioned in 100 mM Tris-HCl buffer solution pH 7.4 at 4 °C for 24 h. Likewise, the electrodes were functionalized using larger volume of proteins solution. The 30 µL and 60 µL of urease and BSA solution were deposited on the surface of the electrodes. All other parameters were the same as described above. The studied BSA and urease concentration ratios are presented in Table 1.

**Figure 1:**
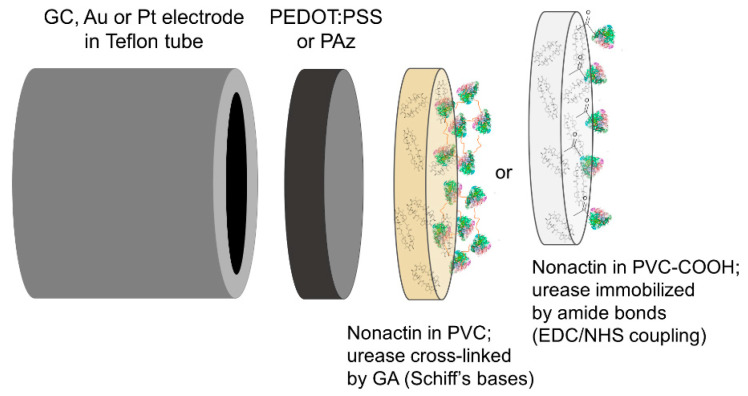
General construction of the urea biosensor.

In the case of PVC-COOH based ion-selective membranes (ISM_II_), covalent attachment of urease was performed by EDC/NHS coupling. Electrodes with ISM_II_ were coated with 60 µL EDC/NHS (40:10 mM:mM) and urease in water (12 mg/mL) and left for 5 h. The excess of enzyme was removed by rinsing with deionized water. Then, biosensors were conditioned in 100 mM Tris-HCl buffer solution of pH 7.4 at 4 °C for 24 h. The construction of urea biosensor is shown in Figure 1. A comparison of urea biosensors’ response for urea concentration step from 1 to 5 mM is shown in Figure S3 in Supplementary materials.

### 2.5. Determination of Enzyme Activity and Michaelis–Menten Constant

The initial slope method was used to determine the urease activity [15]. For urease activity measurements, GC/PAz/ISM_I_ sensors were used. Three laboratory samples containing 37.5, 131.0 and 316.5 U/L in 100 mM TRIS-HCl solution (pH 7.4) were prepared. The 100 mM TRIS-HCl buffer solution of pH 7.4 was found to be optimal, as reported in our previous studies [12,13].

Urea and ammonium chloride were added to the prepared solutions to receive final concentration of 50 mM urea and 0.01 mM ammonium ion, respectively. Before measurement, sensors were calibrated in the concentration range from 0.01 to 100 mM of NH_4_Cl solutions in 100 mM TRIS-HCl at pH 7.4. Each measurement was conducted using 6 sensors at 25 °C. A series of 100 mM TRIS-HCl pH 7.4 solutions was prepared and NH_4_Cl and urea were added to obtain final concentration of 0.01 mM and 50 mM, respectively. The electromotive force (EMF) of ammonium sensors in the prepared solutions was stabilized for 10 min. Then appropriate volumes of urease standard solution (500 U/mL) were added to obtain enzyme concentration from 5 to 1000 U/L. The change of EMF was measured over the next 10 min.

In order to determine the Michaelis–Menten constant, a series of 0.01 mM NH_4_Cl solutions were prepared. Appropriate volumes of 1 M urea standard solution were added to obtain concentrations ranging from 0.1 mM to 30 mM (all solutions based on 100 mM TRIS-HCl at pH 7.4). The EMF recording was started after the ammonium sensors were immersed in the prepared solution. Then, after 10 min, 20 µL of urease standard solution (500 U/L) was added. The rate of the urease-catalyzed reaction was determined as a function of the substrate concentration. Finally, Lineweaver–Burk linearization was used to analytically determine *K_M_* and *V_max_*.

### 2.6. Urea Determination in Saliva Samples

The usability of the potentiometric urea biosensors was confirmed by testing 3 samples of human saliva. Samples were self-collected by a volunteer (one of the authors) in the morning before meals and brushing teeth. Human saliva samples were collected to sterile 5 mL syringes. The collected samples were then placed in Eppendorf vials and centrifuged for 5 min at 6000 RPM to obtain clear saliva samples. The pH of each sample was measured.

In order to minimize the influence of K^+^, the main interferent present in the saliva samples, the following protocol was utilized. The base solutions of 1 mM urea and 1 mM of ammonium ions were prepared in 100 mM TRIS-HCl buffer solution of pH 7.4. To the known volume of each base solution, 0.5 mL of saliva sample was added. The urea concentration was determined 3 times using six biosensors (GC/PAz/ISM_I_/E), while ammonium ion concentration was measured 3 times using six GC/PAz/ISM_I_ sensors simultaneously. The single measurement time for each sample was 10 min. The ammonium ion and urea concentration were calculated based on the EMF change, recorded for the base solutions (1 mM urea and 1 mM NH_4_^+^) before and after saliva sample additions.

A colorimetric method was used as a reference. A commercially available urea kit (QuantiChrom™ Urea Assay Kit), based on an improved Jung’s direct method was used to determine the urea in the tested samples. Following the protocol provided by the supplier, 200 µL of mixed R1 and R2 reagents, together with 5 µL of urea standard (50 mg/dL) or saliva sample, were pipetted into separate wells of 96-well microplate. The optical density was read at 520 nm using microplate spectrophotometer Synergy HT (BioTEK).

Additionally, an improved Berthelot’s method was used to determine the ammonium ion and urea levels. Following Remiszewska et al.’s [33] protocol, two solutions were prepared: S1 composed of 180 mM sodium salicylate, 15 mM sodium nitroprusside and 2 mM EDTA in phosphate buffer solution of pH 7 and reagent S2 containing 16.9 mM sodium hypochlorite in phosphate buffer solution of pH 12. The ammonium ion concentration was determined by pipetting 100 µL of S1 and 1 µL of urea standard solution (50 mg/dL) or saliva samples into separate wells of 96-well microplate (non-enzymatic reaction). After 10 min of incubation at room temperature, 100 µL of S2 was added to each cell. The optical density was read at 700 nm using microplate spectrophotometer Synergy HT (BioTEK) after 10 min of incubation.

Urea determination was performed analogously as it was described for ammonium ions; however, mixing the S1 with 600 kU/L urease solution in 100:1 ratio (enzymatic reaction). The urea present in the sample was hydrolyzed by urease to ammonia and carbon dioxide. The obtained result was therefore the sum of ammonium ions derived from enzymatic urea decomposition and ammonium ions present in the tested samples. The urea concentration was obtained by subtracting the ammonium ions concentration (known from the non-enzymatic assay) from the total ammonium ions concentration obtained in the enzymatic urea assay.

All waste (saliva samples and container being in contact with saliva) was collected into autoclavable bags and autoclaved prior to discard.

## 3. Results and Discussion

### 3.1. Urea Biosensor Based on PEDOT:PSS Electroactive Layer

To produce the urea sensor, GC/PEDOT:PSS/ISM_I_ was biofunctionalized with the urease. Different amounts of urease and BSA listed in Table 1 were used and their influence on biosensor performance was studied. The acceptable sensitivity of the prepared biosensors (above 45 mV/dec) was obtained if enzyme content in the solution was 10 or 12 mg/mL. Increasing the urease concentration to 15 mg/mL did not increase the urea sensor sensitivity. Therefore, the solution containing 12 mg of enzyme per mL was used in further studies. To increase the urease loading on the electrode surface, the volume of protein solution was increased to 60 µL, while the amount of GA solution remained constant. The obtained biosensor, labelled as GC/PEDOT:PSS/ISM_I_/E, showed the highest sensitivity (c.a. 51 mV/dec), and the most favourable limit of detection was equal to 2.2∙10^−5^ M (Table 1).

Although biosensor based on the PEDOT:PSS layer showed Nernstian characteristics, long response time and high signal drift were observed (Table S2). A high value of the signal drift coefficient (~1.7 mV/h) is an unfavourable phenomenon that affects the accuracy of analytical measurements. High signal drift may result from the formation of thin water layer between the PEDOT:PSS layer and ion-selective membrane.

### 3.2. Hydrophobic PAz as Ion-to-Electron Transducing Layer

Although ISEs based on GC/PEDOT:PSS are widely described in numerous scientific reports, such systems are not without drawbacks. They are characterized by the poor reproducibility of standard potential and unsatisfactory long-term potential stability. The main disadvantage of the PEDOT:PSS transducing layer is its hydrophilic character, resulting in a formation of a thin layer of water between polymer surface and ISM, which directly affects the reproducibility of the E^0^ potential and the stability of the sensor. In order to obtain potentiometric sensors with increased stability and sensitivity, we used azulene to manufacture the transducing layer. The azulene was electrochemically polymerized and deposited on the electrode surface by the method described by Lindfor’s group [30,31]. In our experiments, the wettability studies showed that the contact angle of the PEDOT:PSS layer was equal to 47 ± 1° (similarly −48° in ref. [33]), while the PAz layer had a contact angle of 99 ± 1° (98°—after ref. [31]), which confirmed the hydrophobic character of the latter one.

As the sensor stability depends, among others, on the electrode material, three electrodes, namely GC, Au, and Pt were studied. The azulene was electropolymerized on the electronic conductors, and then the layer was coated with the ISM_I_, which was proved in the first stage of this study to possess the best properties for ammonium ions sensor (see Supplementary materials). All sensors were examined in terms of their stability, sensitivity, and selectivity. Both, Au/PAz and Pt/PAz had a similar sensitivity as the GC/PAz sensors. However, low stability (2.2 mV/h nad 2.8 mV/h for Au and Pt electrodes, respectively) and significantly lower selectivity coefficients in relation to the main interfering ion—K^+^; we excluded gold and platinum as electrode material from our further research on the development of a urea sensor. The theory states that selectivity of potentiometric sensor depends on the membrane type. However, it has been observed that the type of underneath conductor layers can also have an impact on sensor behavior, as was reported by Lewenstam group [34]. More recently, Maksymiuk et al. pointed out that different materials and constructions of sensors result in specific conditions of membrane formation. Consequently, an uncontrolled modification of the membrane composition can take place, which could have an influence on membrane selectivity [35].

**Figure 2:**
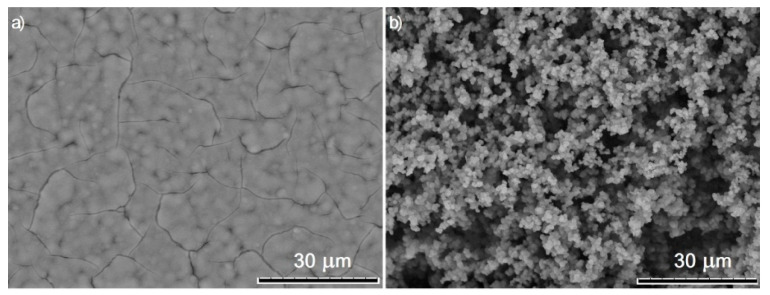
SEM images of (**a**) PEDOT:PSS and (**b**) PAz layers deposited on carbon electrode.

Further investigations were carried out based on the GC/PAz system. The PAz surface deposited on GC was also characterized with electrochemical impedance spectroscopy and scanning electron microscopy (SEM) in comparison to the GC/PEDOT:PSS system. SEM images presented in Figure 2 clearly show the differences in morphology of PEDOT:PSS and PAz layers deposited on carbon electrode. PEDOT:PSS forms uniform, tight layer, while PAz shows rather open, grained structure. It is known that hydrophobic character of the material is extremely dependent on its chemical composition and topography. The chemical structure of polyazulene and the roughness of the created layer makes the surface hydrophobic, which was confirmed by contact angle analysis.

**Figure 3:**
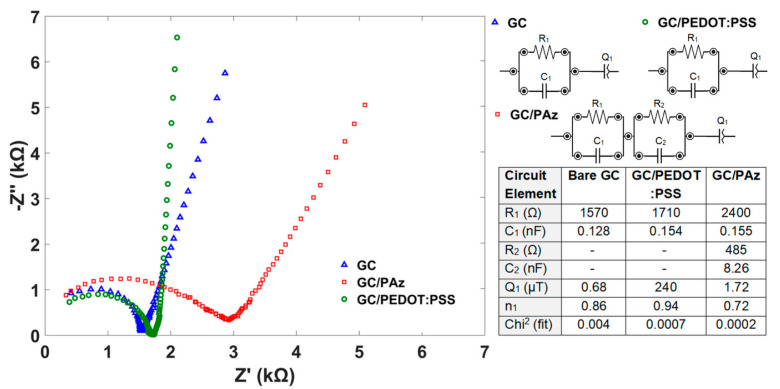
Nyquist plots for bare glassy carbon (GC), GC covered with PEDOT:PSS and PAz conducting polymers, and equivalent circuits (RCQ), and calculated values of the circuit elements (table).

The electrical properties of PEDOT:PSS and PAz interlayers were investigated by means of electrochemical impedance spectroscopy (EIS) method. The measurement data for bare glassy carbon, GC covered with conducting polymers PEDOT:PSS or PAz are presented as Nyquist plots in Figure 3. The equivalent circuits based on the so-called Randles model usually applied for conducting polymers, e.g., for PAz used by Osaka [36], can be considered as applicable for interpretation of impedance data obtained in our experiments. However, the RCQ (resistance, capacitance, and constant phase element) models are converted to an easy numerical fitting of the equivalent circuit in order to estimate value of elements (presented in table inset in Figure 3).

Based on the RCQ equivalent circuits, PAz shows the least capacitive behavior (the lowest *n*_1_—the exponent which defines character of the frequency dependence taking values of 1 ≥ *n* ≥ 0), which may result in improved time stability of the sensor in respect to response and potential *E*^0^. The GC/PEDOT:PSS system exhibits the largest capacitive features, which may explain possible instabilities of the sensor’s response.

The response time (*t*_95%_) of the sensors was defined as the time in which the potentiometric signal reaches 95% of the final value. Compared to the GC/PEDOT:PSS/ISM_I_ electrodes (t_95%_ equal to approx. 45 s), the GC/PAz/ISM_I_ electrodes show c.a. 10-fold decrease of the response time. The signal stability was revealed by measuring the signal drift and determining drift coefficient. The drift coefficient was described as the signal change during 1 h of measurement for the concentration of 1 mM NH_4_^+^. Compared to the GC/PEDOT:PSS electrodes, as expected, the PAz electrodes showed significantly lower signal drift coefficient. The lowest signal drift coefficient was obtained for GC/PAz/ISM_III_. As mentioned before, the surface of PEDOT:PSS modified electrode is hydrophilic in contrast to the surface of the PAz modified electrodes, which are hydrophobic. Due to the hydrophilic nature of PEDOT:PSS, during conditioning, a layer of water forms between the polymer layer and the membrane, which has an influence on the stability of the electrode and increases the potentiometric response time. The hydrophobic nature of the PAz has a positive effect on the stability of the ion-selective electrodes’ response. It stays in agreement with the previously discussed results for highly hydrophobic PEDOT derivative and PAz ion-to-electron transducer.

### 3.3. Urea Biosensor Based on PAz Electroactive Layer

Ion-selective electrodes based on PVC or PVC-COOH membrane with fluorinated lipophilic salt (GC/PAz/ISM_I_ and GC/PAz/ISM_II_) were biofunctionalized with urease by two protocols based on GA crosslinking and EDC/NHS coupling, described in the experimental paragraph, respectively. Following the most efficient protocol employed for GC/PEDOT:PSS/ISM_I_/E, the PAz-based ISEs with PVC membrane were biofunctionalized by dropping 60 µL of urea and BSA solution (12 and 10 mg/mL, respectively) and 30 µL of 5% glutaraldehyde solution. In the case of ISEs with PVC-COOH membranes, biofunctionalization was performed by covalent binding of urease to the carboxylic groups present on the membrane surface using an EDC/NHS coupling agent. Three polymers with 1.8, 5 or 10% of carboxylic groups were tested. Metrological parameter analysis of ammonium ion sensors based on PVC and PVC-COOH has shown that carboxylation may have a beneficial effect on the response time of the sensor. However, the urea sensors with carboxylated PVC showed unsatisfactory sensitivity and signal stability. The lowest sensitivity was obtained for biosensors with covalently bound urease to PVC-COOH with 1.8% of carboxylic basis. Increasing the degree of PVC carboxylation slightly increased the sensitivity of urea biosensor, but these values were significantly lower than the sensitivity of urea sensor based on CG/PAz covered with PVC ion-selective membrane. The GC/PAz/ISM_I_/E urea biosensor with enzyme molecules cross-linked by glutaraldehyde revealed the best sensitivity (52.4 ± 0.7 mV/dec), short response time (t_95%_ = 36 s), and also high stability of the potentiometric response, with drift coefficient two times lower than for analogous sensor with PEDOT:PSS transducer. The differences in the sensitivity of characterized urea biosensors result primarily from the amount of immobilized enzyme, which affects the amount of ammonium ions produced per unit of time. The highest enzyme loading was obtained when glutaraldehyde was used. In the case of PVC-COOH membrane, the enzyme immobilization was limited by number of carboxylic groups present on the surface. Moreover, the usage of PVC-COOH membrane had a positive effect on the response time of ammonium ion sensors, probably due to the electrostatic interaction between negatively charged surface and positively charged ammonium cations. However, after enzyme coupling, carboxyl groups were converted to amides, hindering the electrostatic interaction between the membrane and solution components. Comparison of the metrological parameters and calibration curves recorded for studied urea biosensors are shown in Table 2 and Figure S2, respectively.

As in the case of ammonium sensors, stabilization of the signal is faster for biosensors based on GC/PAz than for GC/PEDOT:PSS, which reflects in lower the drift coefficient and t_95%_ (Table 3). In the case of urea biosensors where a carboxylated ion-selective membrane with covalently bound enzyme was used, next to the non-Nernstian response, one can see high instability of the signal, which resulted in a high drift coefficient value. The response characteristics of different urea biosensors are shown in Supplementary materials Figure S3.

**Figure 4:**
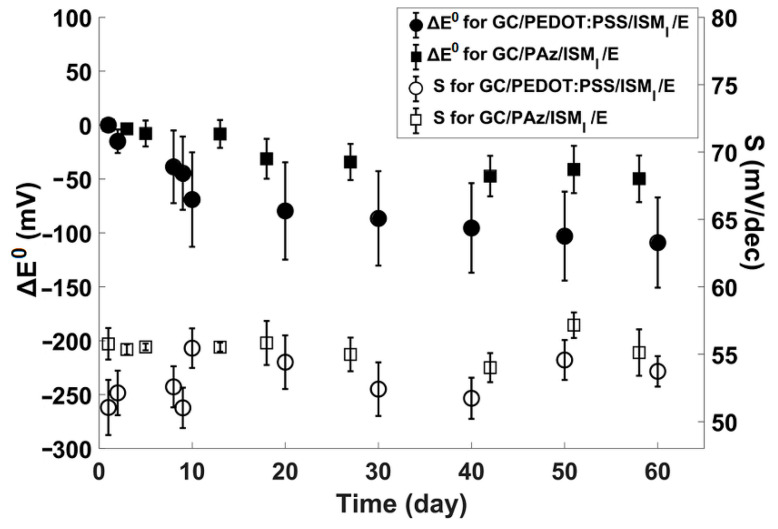
Comparison of ΔE^0^ and sensitivity (S) long-term stability of biosensors with PEDOT:PSS and PAz solid-contacts.

For practical usage, constructed biosensors should be reproducible and stable for required time. The effectiveness of using hydrophobic PAz as an ion–electron transducing layer in stabilizing E^0^ was proven by examination of six electrodes for 60 days. As can be seen in Figure 4, reproducibility and long term stability among different GC/PAz electrodes was much higher as compared to GC/PEDOT:PSS-based biosensors. In particular, the E^0^ variability of GC/PEDOT:PSS-based biosensors are clearly evidenced in Figure 4. The changes of the sensitivity in time, for both, PEDOT:PSS and PAz-based biosensors, are less pronounced. However, sensors with a PAz layer showed higher sensitivity throughout the testing period.

The results of the above-described experiments proved that the most favourable metrological parameters were obtained for the urea biosensor, which was based on a GC electrode coated with PAz with an ion-selective membrane containing a fluorinated lipophilic salt (GC/PAz/ISM_I_/E).

### 3.4. Determination of Urease Activity

In biological and medical research, enzymatic activity is a useful parameter to examine metabolic pathways and their disorders. Measurement of urease activity can be an excellent parameter for determining the bacterial activity or disease marker. On the other hand, urease is used as bioreceptor in a number of assays. In the case of biosensing, the enzyme activity is of great importance and there is a need for a fast and reliable method for its determination. It is not frequently discussed that activity of an enzyme, which serves as a bioreceptor, may change depending on the environment in which it is operating. Responding to these needs, a highly stable potentiometric sensor was developed and tested. Ammonium GC/PAz/ISM_I_ sensors were used to determine the urease activity in the laboratory samples. The ammonium ions created during the enzymatic reaction generate the EMF change, which can be converted to concentration using a calibration curve for urea biosensor (Figure S2).

**Figure 5:**
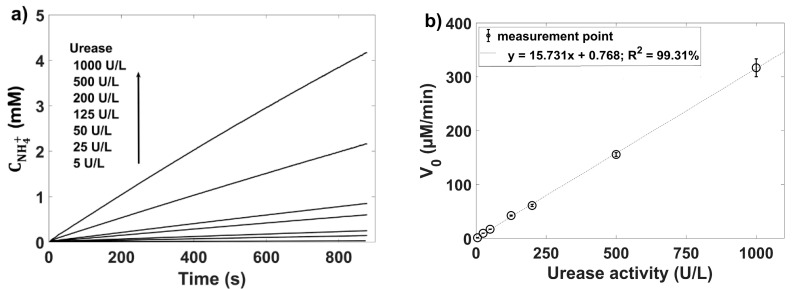
(**a**) Kinetics of urea decomposition reaction at constant initial urea concentration for given urease activity (5–1000 U/L) and (**b**) corresponding calibration curve plotted as dependence between initial enzymatic reaction rate (*V*_0_) and urease activity.

Figure 5a presents kinetics of urea decomposition reaction at constant initial urea concentration for given urease activity (5–1000 U/L) and corresponding calibration curve plotted as initial enzymatic reaction rate (*V*_0_ = *V_max_*∙[*S*]/(*K_M_* + [*S*])) as a function of urease activity. The dependence shown in Figure 5b was obtained by initial slope method [15], which means that the increase of ammonium ions concentration during 1 min was determined for each urease addition.

The GC/PAz/ISM_I_ ammonium ion sensors were used to determine urease activity in three prepared laboratory samples containing 37.5, 135.0 or 316.5 U/L of urease, respectively. The results are presented in Table 3 and compared with the expected values.

In the literature concerning urease activity determination, NH_4_^+^ selective electrodes have been sparsely reported. In the studies presented by Katz [7], cationic sensitive glass electrode was used to determine urease activity in the laboratory samples. The sensitivity of 50.1 mV/dec and long response time (5 min) was observed. Activity of 20–50 mmol NH_3_/g urease was reported in the paper; however, there is no information about the urease source and units/g solid. A classic ammonium ion selective electrode with internal electrolyte and liquid membrane based on plasticized poly(vinyl chloride) containing physically immobilized nonactin as an NH_4_^+^-ionophore and a lipophilic salt was used by Krajewska et al. [37]. The studied sensor revealed sensitivity of 55–57 mV/dec and linear range from 0.0025 to 9.1 mM NH_4_^+^; however, the observed stability and reproducibility at low NH_4_^+^ concentrations was rather poor, hence it was used for kinetic studies under different experimental conditions but not to enzyme activity measurements.

**Figure 6:**
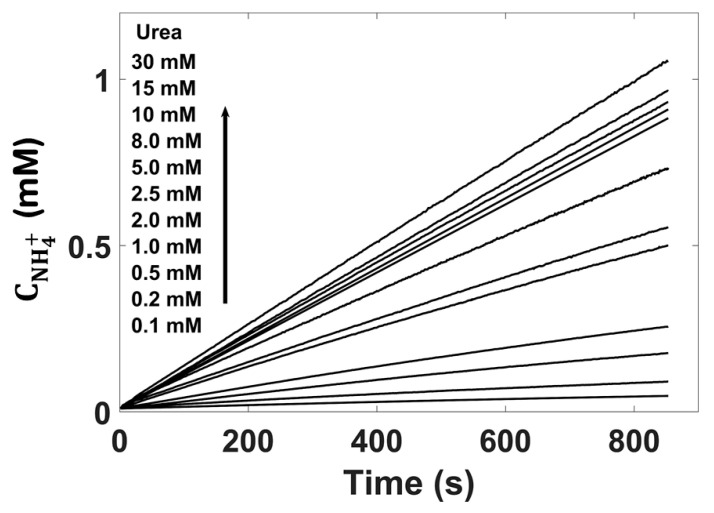
Kinetics of urea decomposition at constant urease activity (500 U/L) and different initial concentrations of urea (0.1–30 mM).

The next stage of the study involved the determination of the Michaelis–Menten constant (K_M_) for the urease–urea reaction. The experiment was carried out using six GC/PAz/ISM_I_ ammonium sensors at constant enzyme activity of 500 U/L, varying the urea concentration from 0.1 to 30 mM. Kinetics of urea decomposition, i.e., the change of concentration of ammonium ions plotted vs. time is shown in Figure 6.

**Figure 7:**
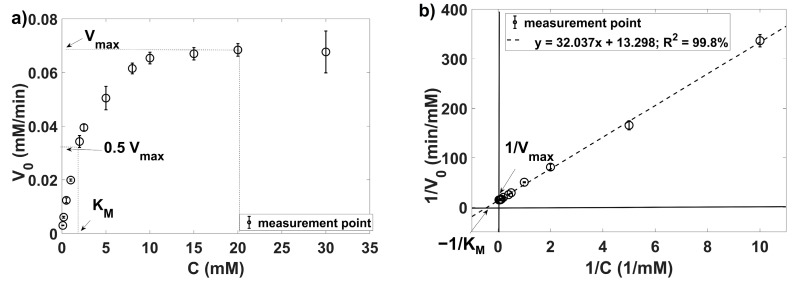
(**a**) Dependence of initial reaction rate (*V*_0_) and urea concentration and (**b**) Lineweaver–Burk plot.

In accordance with generally accepted principles, the rate of the enzymatic reaction depends also on the concentration of the substrate. Based on the results presented in Figure 6, the initial enzymatic reaction rate (V_0_) was plotted against urea concentration and depicted in Figure 7a. The Lineweaver–Burk linearization of curve presented in Figure 7a was used to analytically determine *K_M_* and *V_max_* value (Figure 7b). The slope of the linear function describes the relationship between *K_M_* and *V_ma_*_x_ (tan(*α*) = *K_M_*/*V_max_*). The intersection point with the ordinate axis determines the 1/*V_max_* value and 1/*K_M_* with the abscissa axis.

The K_M_ value for urea–urease reaction obtained in our study was equal to 2.4 mM (±0.11 mM), which corresponds well to the values presented in the literature, where *K_M_* is in the range from 1 to 4 mM [37,38]. The optimal temperature and pH for Jack bean urease catalytic activity is 25 °C and pH 7–8. Decrease of enzyme affinity was reported for temperatures higher than 37 °C and pH outside the range 5.3–9.1 [39]. In our studies, the calculated *K_M_* value is in the mid of the *K_M_* range, which means that the overall experimental conditions do not affect, neither the enzymatic conversion of urea in the presence of urease, nor GC/PAz/ISM_I_ sensor response. In other words, the *K_M_* of 2.4 mM for urease proves that an enzyme affinity for its substrate (urea) was preserved under specific experimental conditions, indicating that the measured activity of the enzyme is reliable.

### 3.5. Testing of Developed Sensors in Saliva Samples

The newly developed biosensor based on the PAz transducing layer was used to determine urea in human saliva samples. Human saliva is the readily available and often underestimated, non-invasively accessible body fluid that may be of diagnostic importance. Urea is a significant component of saliva, as it plays an important role in maintaining the health of oral cavity and teeth. Many plaque bacteria possess urease enzyme, which catalyzes the hydrolysis of urea to ammonia and carbon dioxide. The ammonia, being highly alkaline, neutralizes acids and causes pH rise, which may be a major impediment to the development of dental caries [40]. Moreover, since a high level of nitrogen compounds in saliva reflects their high concentration in blood plasma, it can be used to control the general condition of patients.

Human saliva samples were collected from one of the authors and prepared as described in the Section 2.5. Due to the presence of urea and ammonium ions in saliva, differential measurement with the urea biosensor and ammonium ion sensor was performed. Simultaneously six ammonium GC/PAz/ISM_I_ sensors and next, six GC/PAz/ISM_I_/E biosensors for urea were simultaneously used to measure ammonium ions (*C_A_*) and the total concentration of ammonium ions (*C_U+A_*) in the saliva samples, respectively.

As a reference, spectrophotometric analysis based on both an enzymatic Berthelot’s method and non-enzymatic Jung’s method (QuantiChrom urea kit) was conducted. The Jung’s reaction is specific for urea, and the result is unaffected by ammonium ions. On the other hand, Berthelot’s method was invented to determine ammonia/ammonium ions; however, its enzymatic version can be used to quantify the level of urea. First, the Berthelot’s method was used to determine ammonium ions in the saliva samples. In enzymatic Berthelot’s analysis, urea present in the sample is hydrolyzed by urease to ammonium ions and carbon dioxide. Therefore, finally the sum of ammonium ions present in the sample and ammonium ions produced in the enzymatic reaction is measured. In this respect, the principle of Berthelot’s assay is analogous to the potentiometric approach. The results are compared in Table 4.

It is clear that urea levels determined by potentiometric biosensors are in line with the urea concentrations obtained by both colorimetric methods. The urea concentrations obtained by means of potentiometric analysis and QunatiChrom™ urea test (Jung’s method) differ by c.a. 3% (Δ*_P−J_*), proving high accuracy of potentiometric measurements. The differences between the potentiometric and Berthelot methods (Δ*_P−B_*) are greater; however, by considering the Berthelot vs Jung method, it was found that there was a greater discrepancy between the two reference methods than between the potentiometric method and the Jung method (Δ*_P−J_*). This may be due to the fact that the spectrophotometric determination of urea by the Berthelot method relates to the reaction taking place in the entire sample volume, while in the case of potentiometric sensors, the enzymatic reaction is local, in the vicinity of the electrode surface, which results in faster equilibrium.

## 4. Conclusions

A highly stable potentiometric biosensor based on the GC/PAz system was developed for determination of urea and the enzymatic activity of urease. The study of the individual sensor elements allowed for the development of the optimal composition of the ion-selective membrane, the biofunctionalization solution, and the selection among Au, Pt, and GC substrates of the glassy carbon as a material for solid-contact electrode and the conductive polymer. The selection of PAz as an ion-to-electron transducing layer was based on an excellent stability of sensors, resulting from their electrical properties investigated by means of electrochemical impedance spectroscopy. Studies have shown that: (1) the use of PVC with various levels of carboxylation was not effective for biofunctionalization of the ion-selective membrane—too low concentration of the enzyme at the membrane surface results in low sensitivity of the biosensor; (2) the use of a hydrophobic PAz conducting polymer as an ion-to-electron transducing layer enables obtaining more stable signal compared to the analogically modified electrode by the hydrophilic PEDOT:PSS; and (3) for PAz modified gold and platinum electrode substrates, a high signal drift was observed for the tested ISEs.

The developed highly stable potentiometric ammonium sensor (GC/PAz/ISM_I_) allowed not only to effectively determine the kinetic parameters of the enzymatic reaction catalyzed by urease but also to determine urease activity in laboratory samples. Moreover, the GC/PAz/ISM_I_ ammonium sensor biofunctionalized with urease enabled the measurement of urea concentration in real human saliva samples, which indicates that the new highly stable urea biosensor presented in this paper may be useful in biomedical measurements.

## Figures and Tables

**Table 1:** Comparison of basic metrological parameters for urease-modified sensors, (measurements repeated 3 times for each group of six sensors, n = 3).

Urease:BSA (mg:mg in 1 mL)	5:5	10:10	12:12	7:5	10:7	12:10	12:10
V (μL)	30						60
Sensitivity (mV/dec)	32.8 ± 4.9	45.4 ± 2.1	46.1 ± 2.5	42.9 ± 2.1	46.4 ± 0.8	46.7 ± 0.7	51.4 ± 1.4
LOD (M)	3.6∙10^−4^	7.9∙10^−5^	3.9∙10^−5^	5.1∙10^−5^	5.1∙10^−5^	4.0∙10^−5^	2.2∙10^−5^

**Table 2:** Metrological parameters of studied urea biosensors.

Biosensor	S (mV/dec)	LOD (M)	t_95%_ (s)	Drift Coefficient (mV/h)
GC/PAz/ISM_I_/E	52.4 ± 0.7	1.6∙10^−5^	36	~0.9
GC/PAz/ISM_II_(1.8%-COOH)-E	39.9 ± 2.2	5.1∙10^−5^	74	~5.7
GC/PAz/ISM_II_(5%-COOH)-E	40.9 ± 0.9	2.8∙10^−5^	40	~6.1
GC/PAz/ISM_II_(10%-COOH)-E	43.9 ± 2.5	7.1∙10^−5^	35	~8.3
GC/PEDOT:PSS/ISM_I_/E	51.4 ± 1.4	2.2∙10^−5^	43	~1.7

**Table 3:** Urease activity in laboratory samples determined by six GC/PAz/ISM_I_ potentiometric sensor, with n = 3 repetitions.

Urease Activity Standards (U/L)	Measured Urease Activity by Potentiometric Sensor (U/L)	Δ (%)
36.5	36.9 ± 1.2	1.1
131.0	129.6 ± 2.1	−1.1
316.5	322.3 ± 1.8	1.8

**Table 4:** Results of determination of ammonium and urea in human saliva by potentiometric sensors in reference to colorimetric methods (Δ = (*C* − *C_Ref_*)/*C_Ref_*).

Sample	*C_A_* (mM)		
	ISE_NH4+_ (mean ± SD)	Berthelot’s reaction (mean ± SD)	Δ (%)
P1	14.31 ± 0.14	14.30 ± 0.40	0.07
P2	7.68 ± 0.18	7.74 ± 0.27	−0.78
P3	9.60 ± 0.05	9.92 ± 0.30	−3.33
Sample	*C_A+U_*_,_ (mM)		
	Urea biosensor (mean ± SD)	Enzymatic method based on Berthelot’s reaction (mean ± SD)	Δ (%)
P1	17.76 ± 0.23	18.10 ± 0.23	−1.91
P2	13.19 ± 0.09	13.81 ± 0.41	−4.70
P3	15.93 ± 0.20	16.64 ± 0.11	−4.46
Sample	*C_U_* (mM)		
	Potentiometric method ${\bar{C}}_{U}={\bar{C}}_{A+U}-{\bar{C}}_{A}$ (mM)	Berthelot’s methods ${\bar{C}}_{U}={\bar{C}}_{A+U}-{\bar{C}}_{A}\;$(mM)	Δ*_P−B_* (%)
P1	3.45 ± 0.27	3.80 ± 0.46	−9.21
P2	5.50 ± 0.20	6.07 ± 0.49	−9.40
P3	6.33 ± 0.21	6.72 ± 0.32	−5.80
	Δ*_P−J_* (%)	*C_U_* (mM) Jung’s method (QunatiChrom™) (mean ± SD)	Δ*_B−J_* (%)
P1	−2.90	3.55 ± 0.08	7.04
P2	−3.45	5.69 ± 0.06	6.68
P3	−3.48	6.55 ± 0.09	2.60

## Supplementary Materials

The following are available online at https://www.mdpi.com/article/10.3390/membranes11110898/s1, Figure S1: Calibration curves of ammonium ions sensors with various ion-selective membranes (ISM_I_ and ISM_II_ based on PVC and PVC-COOH, respectively) and transducing layers, as PAz or PEDOT:PSS deposited on Pt, Au and GC electrodes. Figure S2: Calibration curves for studied urea biosensors based on (a) PVC ion-selective membranes deposited on PEDOT:PSS or PAz and (b) PVC-COOH ion-selective membranes deposited on PAz. Figure S3: Comparison of urea biosensors response for concentration step from 1 to 5 mM of urea. Table S1: Compositions of ion-selective membrane cocktails. Table S2: Comparison of ammonium-selective sensors metrological parameters based on PEDOT:PSS and PAz transducing layer.
