# Supplementary material for: Highly Stable Potentiometric (Bio)Sensor for Urea and Urease Activity Determination
Source: Membranes (Basel). 2021 Nov 20;11(11):898. doi: 10.3390/membranes11110898 (PMC8623495; doi:10.3390/membranes11110898)
Supplement: Supplementary file 1 [file membranes-11-00898-s001.zip › membranes-1447352-supplementary.pdf]

membranes

Article

Highly Stable Potentiometric (Bio)Sensor for Urea and Urease Activity Determination

Marcin Urbanowicz *, Kamila Sadowska, Agnieszka Paziewska-Nowak, Anna Sołdatowska and Dorota G. Pijanowska

Nalecz Institute of Biocybernetics and Biomedical Engineering, Polish Academy of Sciences, Ks. Trojdena 4, 02-109 Warsaw, Poland; ksadowska@ibib.waw.pl (K.S.); apaziewska@ibib.waw.pl (A.P.-N.); asoldatowska@ibib.waw.pl (A.S.); dpijanowska@ibib.waw.pl (D.G.P.)

* Correspondence: murbanowicz@ibib.waw.pl

Ammonium ion-selective electrodes with four different membrane composition listed in Table S1 were prepared. Manufactured ISEs were compared in terms of metrological parameters such as sensitivity, selectivity and linear range. The results are summarized in Table S2.

In the first stage, three variants of ion-selective membrane composition (**Table S1**, ISM_I, ISM_{III} and ISM_{IV}), deposited on ion-to-electron PEDOT:PSS transducing layer were studied. Regardless if chlorinated or fluorinated lipophilic salt, as well as no lipophilic salt was used, all studied ISEs exhibited Nernstian response. The selectivity coefficients were determined by both, separate solution method (SSM) and fix interference method (FIM), and they are listed in **Table S2**. Obtaining the highest selectivity in respect to K^+ and Na^+ is important due to the high concentration of these ions in biological samples, including human saliva, which was discussed in our previous studies. Among PEDOT:PSS-based sensors, GC/PEDOT:PSS/ISM_I showed the most favourable selectivity coefficients to key interfering ions, equal to -1.36 for K^+ and -2.82 for Na^+ ions, respectively. Small addition of lipophilic salt into the ion-selective membrane allowed to reduce impedance of the membrane and to improve the co-extraction of ammonium ions from the analysed solution. Additionally, a lipophilic anions prevent the formation of ion pairs on the membrane surface and improves the sensor response.

Next to PVC membrane with fluorinated lipophilic salt, analogous membrane based on PVC-COOH was investigated (**Table S2**, ISM_{II}). The polymers of three levels of functionalisation (1.8%, 5% and 10% of carboxylic basis) were compared. The implementation of carboxylated PVC was driven by the possibility of simple bioreceptor immobilization by means of typical peptide bonding. Calibration curves registered for PAz-based ammonium ISEs in comparison to GC/PEDOT:PSS/ISM_I ammonium sensor are shown in **Figure 1S**. All calibration curves presented in **Figure 1S** have a Nernstian character.

Metrological parameters of all studied ammonium-selective electrodes are summarised in **Table 2S**. It can be seen, that all characterized ISEs showed Nernstian slope and linear range between 10^{-1} - 10^{-5}. The substantial differences between proposed construction, were however observed in the stability and the response time.

Table S1. Compositions of ion-selective membrane cocktails.

Membrane ingredients	ISM_I m/m %	ISM_{II}(X% COOH) m/m %	ISM_{III} m/m %	ISM_{IV} m/m %
**Nonactin**	1	1	1	1
**KTpClPhB**	-	-	-	0.2
**KTpFPhB**	0.2	0.2	-	-
**DOS**	66.8	66.8	66.8	66.8
**PVC**	32	-	32.2	32
**carboxylated PVC**	-	32	-	-

KTpClPhB - potassium tetrakis(p-chlorophenyl)borate; **KTpFPhB -** potassium tetrakis[3,5-bis(trifluoromethyl)phenyl]borate**;** **DOS -** bis-(2-ethylhexyl)-sebacate**;** **PVC -** poly(vinyl chloride); **X -** 1.8%, 5% or 10% carboxyl basis

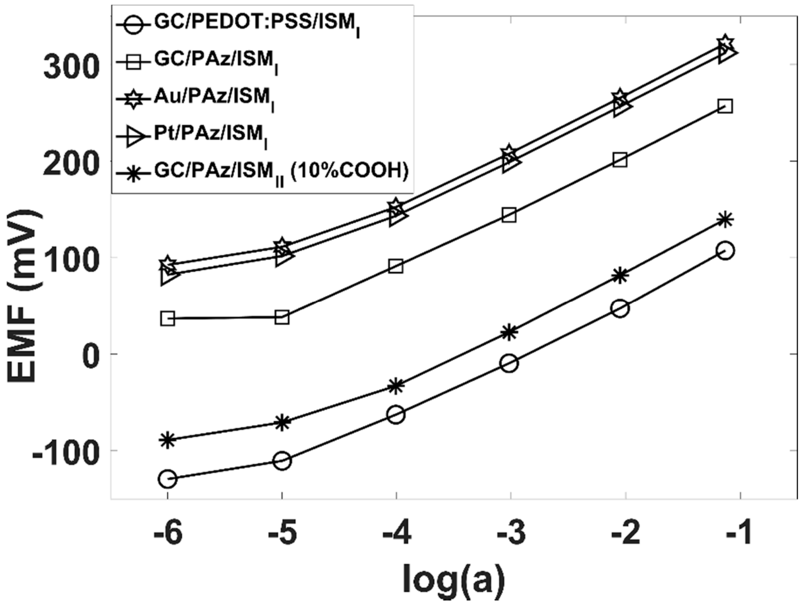

Figure S1. Calibration curves of ammonium ions sensors with various ion-selective membranes (ISM_I and ISM_{II} based on PVC and PVC-COOH, respectively) and transducing layers, as PAz or PEDOT:PSS deposited on Pt, Au and GC electrodes.

Table S2. Comparison of ammonium-selective sensors metrological parameters based on PEDOT:PSS and PAz transducing layer.

**Parameter**	**GC/PEDOT:PSS/ISM$_I$**		**Au/PAz/ISM$_I$**		**Pt/PAz/ISM$_I$**		**GC/PEDOT:PSS/ISM$_{III}$**		**GC/PEDOT:PSS/ISM$_{IV}$**	
	**SSM**	**FIM**	**SSM**	**FIM**	**SSM**	**FIM**	**SSM**	**FIM**	**SSM**	**FIM**
$\log K^{pot}_{NH_4^+/K^+}$	-1.36±0.09	-1.27±0.12	-1.06±0.01	-0.69±0.05	-1.06±0.01	-0.57±0.05	-0.92±0.12	-1.17±0.12	-0.97±0.08	-1.2±0.1
$\log K^{pot}_{NH_4^+/Na^+}$	-4.03±0.10	-2.82±0.10	-3.81±0.01	-3.02±0.03	-3.81±0.01	-3.19±0.04	-3.41±0.09	-3.29±0.09	-3.53±0.11	-3.4±0.1
$\log K^{pot}_{NH_4^+/Ca^{2+}}$	-4.32±0.12	-3.31±0.09	-4.46±0.01	-3.64±0.03	-4.47±0.01	-3.37±0.05	-4.86±0.14	-3.88±0.14	-4.54±0.13	-3.0±0.1
$\log K^{pot}_{NH_4^+/Mg^{2+}}$	-4.66±0.12	-3.32±0.11	-4.44±0.01	-3.78±0.05	-4.45±0.01	-3.91±0.02	-4.18±0.14	-2.98±0.13	-4.49±0.13	-3.2±0.1
$\log K^{pot}_{NH_4^+/Mn^{2+}}$	-3.68±0.14	-2.61±0.11	-4.41±0.01	-3.16±0.12	-4.40±0.01	-3.45±0.02	-3.61±0.10	-2.51±0.11	-3.61±0.10	-2.6±0.1
LR **(M)**	10^{-1} - 10^{-5}		10^{-1}-10^{-5}		10^{-1}-10^{-5}		10^{-1} - 10^{-5}		10^{-1} - 10^{-5}	
LOD **(M)**	$9.1 \cdot 10^{-6}$		$2.7 \cdot 10^{-5}$		$2.6 \cdot 10^{-5}$		$4.5 \cdot 10^{-5}$		$1.2 \cdot 10^{-5}$	
S **(mV/dec)**	56.41±1.36		55.14±0.15		56.12±0.11		56.61±0.23		56.25±0.31	
**$t_{95\%}$ (s)**	42		5		20		47		44	
**Drift coefficient (mV/h)**	~0.5		~2.2		~2.8		~0.8		~0.6	

**Parameter**	**GC/PAz/ISM$_I$**		**GC/PAz/ISM$_{II}$ (1.8%-COOH)**		**GC/PAz/ISM$_{II}$ (5%-COOH)**		**GC/PAz/ISM$_{II}$ (10%-COOH)**		**Liquid internal solution electrode [55]**
	**SSM**	**FIM**	**SSM**	**FIM**	**SSM**	**FIM**	**SSM**	**FIM**	-0.9
$\log K^{pot}_{NH_4^+/K^+}$	-1.28±0.03	-1.22±0.01	-1.11±0.02	-1.19±0.01	-1.11±0.02	-1.19±0.01	-1.11±0.01	-1.19±0.01	-2.9
$\log K^{pot}_{NH_4^+/Na^+}$	-3.89±0.01	-2.97±0.01	-3.18±0.04	-2.52±0.01	-3.18±0.04	-2.52±0.01	-3.25±0.11	-2.92±0.01	-5.0
$\log K^{pot}_{NH_4^+/Ca^{2+}}$	-4.68±0.04	-3.64±0.01	-4.47±0.05	-3.60±0.02	-4.47±0.05	-3.60±0.02	-4.28±0.04	-3.60±0.01	-2.9
$\log K^{pot}_{NH_4^+/Mg^{2+}}$	-4.76±0.07	-3.71±0.01	-4.48±0.08	-3.62±0.01	-4.48±0.08	-3.62±0.01	-4.49±0.06	-3.66±0.01	–
$\log K^{pot}_{NH_4^+/Mn^{2+}}$	-3.82±0.09	-2.97±0.01	-2.75±0.10	-2.93±0.01	-2.75±0.10	-2.93±0.01	-3.08±0.09	-2.93±0.01	–
LR **(M)**	10^{-1}-10^{-5}		10^{-1}-10^{-5}		10^{-1}-10^{-5}		10^{-1}-10^{-5}		10^{-1} -10^{-3}
LOD **(M)**	$1.05 \cdot 10^{-5}$		$3.95 \cdot 10^{-5}$		$1.61 \cdot 10^{-5}$		$3.96 \cdot 10^{-5}$		–
S **(mV/dec)**	56.49±0.09		56.99±1.84		57.53±0.13		55.30±0.15		58.3
**$t_{95\%}$ (s)**	6		3		14		5		38
**Drift coefficient (mV/h)**	~0.2		~0.3		~0.6		~0.4		–

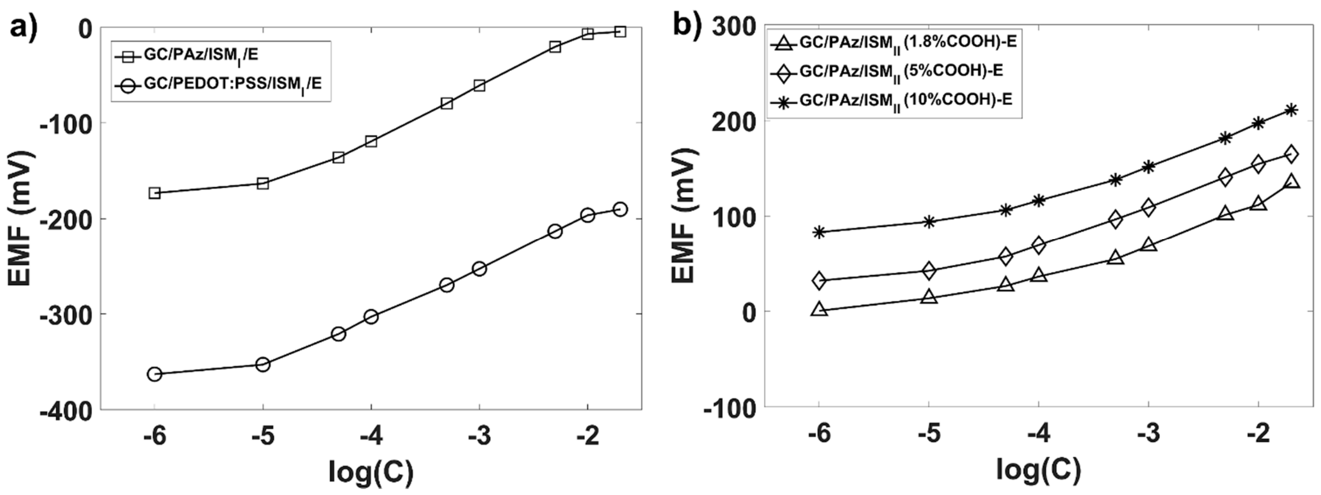

Figure S2. Calibration curves for studied urea biosensors based on (**a**) PVC ion-selective membranes deposited on PEDOT:PSS or PAz and (**b**) PVC-COOH ion-selective membranes deposited on PAz.

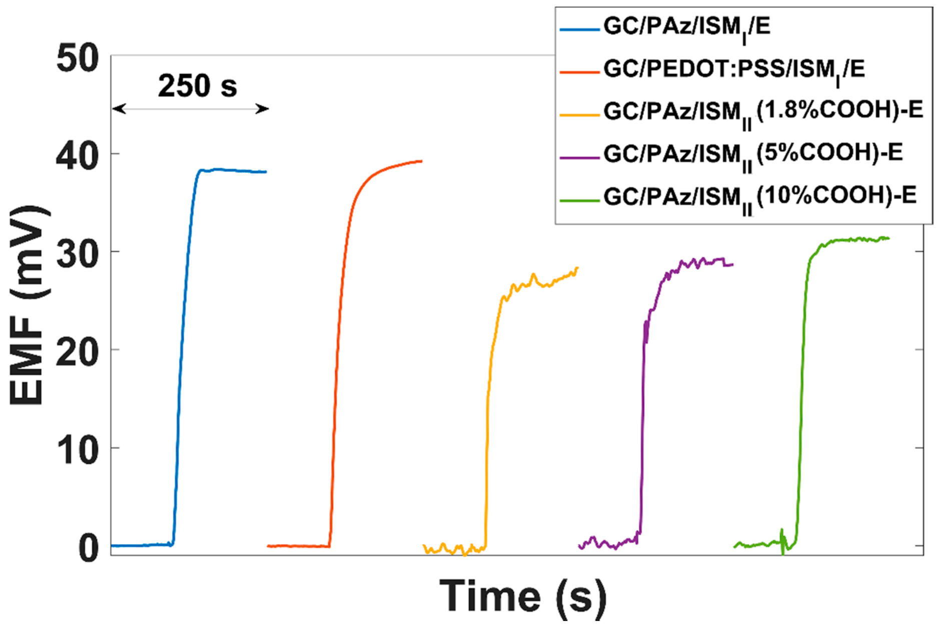

Figure S3. Comparison of urea biosensors response for concentration step from 1 to 5 mM of urea.
